# *Aspergillus fumigatus*-induced acute fibrinous and organizing pneumonia complicated by spontaneous subcutaneous and mediastinal emphysema: a case report and literature review

**DOI:** 10.3389/fmed.2026.1901693

**Published:** 2026-08-17

**Authors:** Qiang-zhong Pi, Min Wu, Hu Luo

**Affiliations:** 1 Department of Respiratory and Critical Care Medicine, The First Affiliated Hospital of Army Medical University (Southwest Hospital), Chongqing, China; 2 Xifeng County People’s Hospital of Guiyang City, Guiyang, Guizhou, China

**Keywords:** acute fibrinous and organizing pneumonia (AFOP), *Aspergillus fumigatus*, case report, literature review, mediastinal emphysema, spontaneous subcutaneous emphysema

## Abstract

**Background:**

Acute fibrinous and organizing pneumonia (AFOP) is a rare and distinct histopathological pattern of acute lung injury characterized by intra-alveolar fibrin deposition. It poses significant diagnostic and therapeutic challenges due to its heterogeneous etiologies and the lack of standardized clinical guidelines.

**Case presentation and methods:**

We report the clinical course of a 58-year-old male presenting with *Aspergillus fumigatus*-associated AFOP complicated by spontaneous subcutaneous and mediastinal emphysema. Diagnosis was confirmed via CT-guided percutaneous lung biopsy. Additionally, we conducted a systematic literature review of PubMed and EMBASE databases (January 2019 to July 2024) to analyze the clinical-radiological features, etiological spectrum, and treatment outcomes of 31 unique AFOP cases.

**Results:**

The case patient presented with fever, chest pain, and dyspnea. Following confirmation of *A. fumigatus*-induced AFOP and management of secondary mediastinal emphysema via negative-pressure closed thoracic drainage, the patient showed a favorable response to combined corticosteroid and antifungal therapy. The literature review of 31 patients revealed a male predominance (17/31, 54.8%) with a mean age of 55 ± 15 years (range: 22–78). Fever was the most prevalent clinical presentation (23/31, 74.2%), followed by cough (19/31, 61.3%) and dyspnea (13/31, 41.9%). Chest computed tomography (CT) predominantly revealed consolidation (22/31, 71.0%) and ground-glass opacities (12/31, 38.7%). Identified etiologies were highly heterogeneous, including idiopathic forms, infections, hematologic malignancies, and autoimmune disorders. Glucocorticoid-based regimens achieved an overall treatment response rate exceeding 73%.

**Conclusion:**

AFOP is a multifaceted pulmonary disorder associated with non-specific symptoms and radiological features like consolidation and ground-glass opacities. Aspergillus infection can trigger AFOP, which may be rarely complicated by spontaneous mediastinal emphysema. Early histopathological confirmation and individualized immunomodulatory therapy are crucial for favorable clinical outcomes.

**Limitations:**

This study is limited by a small literature cohort size, a lack of multivariable analysis, and limited molecular epidemiological data.

## Introduction

Acute fibrinous and organizing pneumonia (AFOP) was first described as a distinct histopathological pattern of acute lung injury in 2002 by Beasley et al. (1). The histological hallmark of AFOP is the intra-alveolar accumulation of fibrin, forming classic “fibrin balls” within the alveolar spaces, accompanied by a patchy distribution of organizing pneumonia. This characteristic pattern lacks the hyaline membranes typical of diffuse alveolar damage (DAD) and the classic fibroblastic plugs of bronchiolitis obliterans organizing pneumonia (BOOP/OP) (2). AFOP can present as an idiopathic condition or secondary to various clinical triggers, including connective tissue diseases, hematological malignancies, drug toxicities, and infectious pathogens,and a wide spectrum of pathogens—including viruses, bacteria, fungi, and parasites—has been implicated in secondary organizing pneumonia (SOP) and AFOP, reflecting a complex host-pathogen immune response (3). However, to our knowledge, *Aspergillus fumigatus*-induced AFOP complicated by spontaneous subcutaneous and mediastinal emphysema has not been previously reported in the medical literature.

Here, we present a rare case of *A. fumigatus*-induced AFOP in a 58-year-old male who presented with a 10-day history of fever, chest pain, and progressive dyspnea, further complicated by spontaneous subcutaneous and mediastinal emphysema during hospitalization. He achieved complete clinical recovery following targeted corticosteroid and antifungal therapy.

## Case presentation

**Figure 1:**
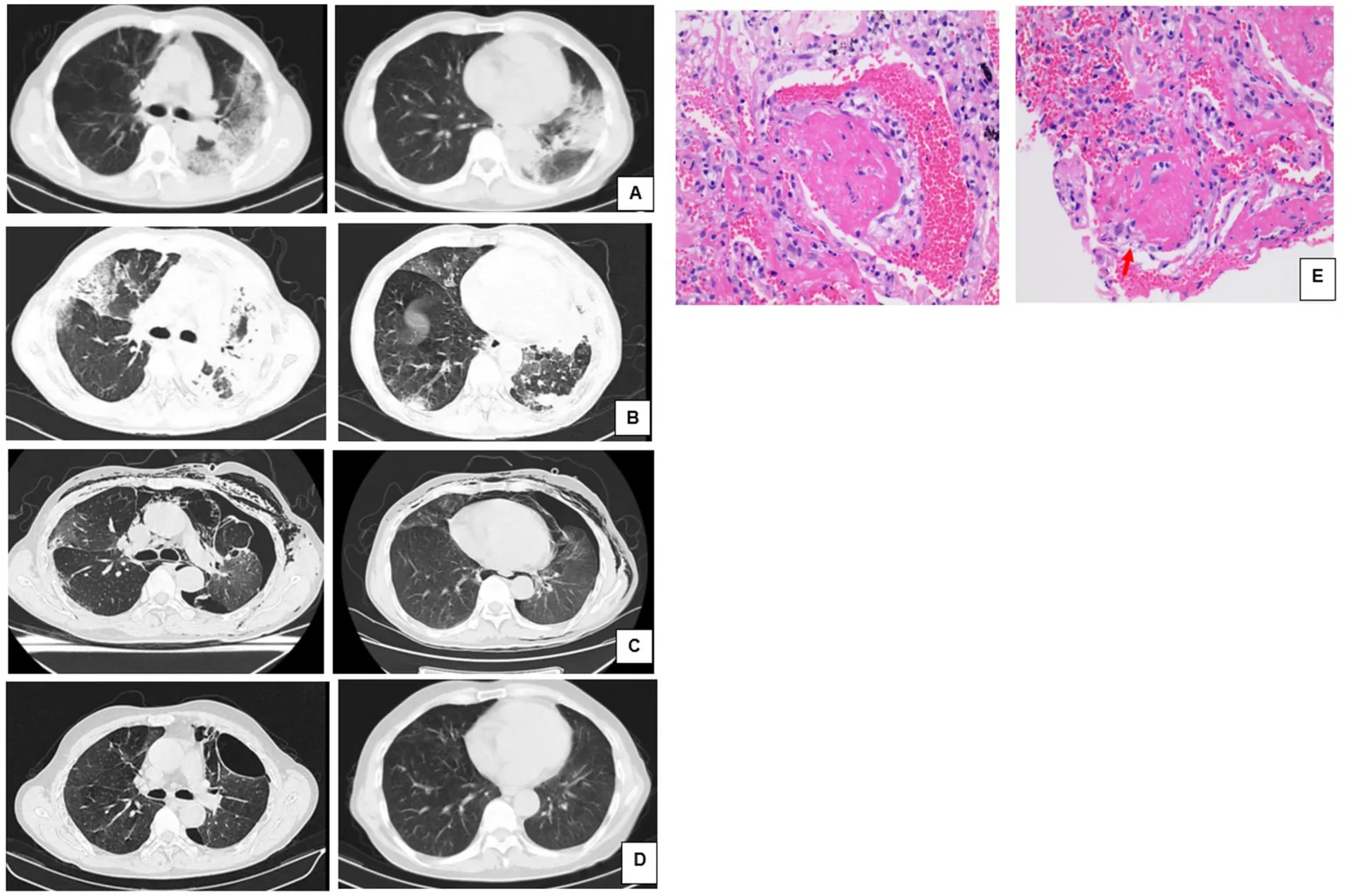
**(A)** Consolidation and ground glass opacity in both lungs, with significant involvement in the left lower lung **(B)** Progression of the pulmonary lesions **(C)** The bilateral pulmonary lesions had absorbed, leaving only a few fibrous strands **(D)** Repeated Chest CT on 23 July 2024 **(E)** Histological examination of CT-guided lung biopsy from right upper lobe cavitary lesion revealed fibrous tissue hyperplasia with fibrinoid exudation and focal deposition of fibrin “balls” in the alveolar cavity (H&E).

A 58-year-old male with no significant past medical, family, or psychosocial history presented to our department with a 10-day history of high fever, chest pain, and progressive shortness of breath. Prior to admission, he had received empirical broad-spectrum antibiotic therapy for suspected community-acquired pneumonia at a local clinic for approximately 2 weeks, with no clinical improvement. Consequently, he was referred to our institution for further diagnostic evaluation. Initial chest computed tomography (CT) revealed bilateral patchy consolidations and ground-glass opacities, with the left lower lobe being predominantly affected (Figure 1A).

Upon admission, physical examination revealed a body temperature of 37.0 °C, heart rate of 116 beats per minute, respiratory rate of 20 breaths per minute, and blood pressure of 91/63 mmHg. Chest auscultation revealed diminished breath sounds with fine crackles in the bilateral lower lung fields. Initial arterial blood gas (ABG) analysis on room air revealed respiratory alkalosis with hypoxemia: pH of 7.51, hypoxemia with a partial oxygen pressure of 63 mmHg, and hypocapnia with a partial carbon dioxide pressure of 29 mmHg. Laboratory results demonstrated an elevated white blood cell count of 11.25 × 10^3^/μL, with neutrophils constituting 85.6%.

On day 4 of admission, cultures from bronchoalveolar lavage fluid (BALF) yielded no bacterial growth. However, the BALF galactomannan (GM) test was positive (index > 0.5), raising suspicion of *Aspergillus* infection. Despite treatment adjustments to Cefoperazone-sulbactam (3.0 g IV q12h) and Omadacycline (100 mg IV q24h), the patient’s condition progressively deteriorated. On the 9th day of admission, laboratory tests revealed the following: white blood cell count 10.58 × 10^9^/L with neutrophil percentage of 79.70%, while procalcitonin (PCT) was 0.20 ng/mL and interleukin-6 (IL-6) was 267.70 ng/L. By day 11, he developed severe type I respiratory failure, and a repeat chest CT demonstrated marked progression of the bilateral pulmonary consolidations (Figure 1B).

To obtain a definitive diagnosis, a CT-guided percutaneous needle lung biopsy of the left lower lobe was performed on day 11. Histopathological examination revealed intra-alveolar fibrin exudation forming characteristic “fibrin balls,” accompanied by type II pneumocyte hyperplasia and mild organizing connective tissue within the alveolar spaces (Figure 1E), confirming a diagnosis of AFOP. Concurrently, *Aspergillus fumigatus* was isolated from the lung tissue biopsy culture, establishing the diagnosis of *A. fumigatus*-associated AFOP.

Based on these findings, the therapeutic regimen was transitioned to targeted antifungal therapy with intravenous voriconazole (0.4 g q12h on day 1, followed by 0.2 g q12h) and anti-inflammatory therapy with methylprednisolone (1 mg/kg/day, 60 mg IV q24h). Within 24 h, the patient’s fever subsided. No glucocorticoid-related adverse events, such as gastrointestinal hemorrhage or hyperglycemia, were observed.

On hospitalization day 24, the patient experienced sudden chest pain and acute dyspnea. A bedside chest CT revealed new-onset spontaneous mediastinal and subcutaneous emphysema (Figure 1C). On the 26th day of hospitalization, re-examination of the complete blood count demonstrated WBC 7.34 × 10^9^/L with neutrophils of 86.70%. Concurrently, interleukin-6, and procalcitonin were all within the normal range. Closed thoracic drainage was promptly initiated. Following systematic optimization of the drainage system with continuous negative pressure suction, the air leak resolved, and the subcutaneous emphysema was completely absorbed.

The patient’s respiratory status steadily improved, and repeat chest CT scans demonstrated resolving pulmonary consolidations (Figure 1C).

On day 56, the patient was discharged in stable clinical condition. His discharge prescription included oral prednisone (20 mg once daily) and oral voriconazole (200 mg twice daily). At his follow-up evaluation on July 23 2024 (on hospitalization day 90), a repeat chest CT demonstrated near-complete resolution of the bilateral consolidations and emphysema (Figure 1D), correlating with complete clinical recovery and high patient satisfaction. Consequently, oral voriconazole was discontinued after 90 days of treatment. Although AFOP is rare, a risk of recurrence has been reported in the literature (1). Therefore, we recommend that patients undergo follow-up chest CT every 6–12 months.

## Literature review

### Methods

This study was approved by the Ethics Committee of the First Affiliated Hospital of Army Medical University (Southwest Hospital, Chongqing, China; Approval No. BIIT2024301). Written informed consent for publication of clinical data and images was obtained from the patient.

To contextualize this case, a systematic literature search was conducted in the PubMed and EMBASE databases for articles published between January 2019 and July 2024. The search string utilized was: (“acute fibrinous and organizing pneumonia” OR “acute fibrinous organizing pneumonia” OR “AFOP”). Only English-language peer-reviewed case reports or series containing detailed individual patient data were included. Duplicate publications, reviews, and articles lacking essential clinical, radiological, or therapeutic data were excluded. All retrieved data were fully anonymized.

## Results

A total of 26 articles describing 31 unique AFOP patients met the eligibility criteria and were included in the cohort analysis (Table 1). Among the 31 patients, there was a slight male predominance (17 males, 14 females), with a mean age of 55 ± 15 years (range: 22–78 years). The clinical onset ranged from acute to subacute. AFOP characterized acute presentations as those with symptom duration of less than 2 weeks, while subacute cases typically evolve over 2 weeks. The most common presenting symptoms were non-specific and primarily respiratory: fever (23/31, 74.2%), cough/sputum production (19/31, 61.3%), and dyspnea (13/31, 41.9%). Extrapulmonary manifestations, such as arthralgia, myalgia, skin erythema, and weight loss, were rarely observed. One patient was completely asymptomatic at presentation. Chest CT imaging findings were highly characteristic of interstitial injury, with consolidations (22/31, 71.0%) and ground-glass opacities (GGOs; 12/31, 38.7%) being the predominant findings. As outlined in Table 2, the etiological spectrum of AFOP was broad and heterogeneous. Identified triggers included idiopathic forms, pulmonary infections, hematological malignancies, connective tissue diseases (CTDs), and adverse drug reactions (particularly to novel oncological immunotherapies).

**Table 1:** Main clinical characteristics and chest CT features of acute fibrinous and organizing pneumonia.

Demographic	Mean ± SD/*n*
Age, years	55 ± 15
Male/*n*	14/31
Symptoms	No. patients, *n* = 31, *n*(%)
Intrapulmonary symptoms	
Cough or sputum	19 (61.3)
Dyspnea	16(51.6)
Chest pain	2 (6.5)
Chest tightness	1 (3.2)
Hemoptysis	1 (3.2)
Extrapulmonary symptoms	
Fever	23 (74.2)
Swelling	3 (9.7)
Erythema	2 (6.5)
Weight loss	2 (6.5)
Anorexia	2 (6.5)
Involvement of the muscular system	2 (6.4)
Polyarthralgia	1 (3.2)
Mouth ulcers	1 (3.2)
Asymptomatic	1 (3.2)
Pulmonary CT imaging findings	No. patients, *n* = 31, *n*(%)
Consolidation	23 (74.2)
Ground-glass opacity	12 (38.7)
Nodules	3 (9.7)
Septal thickening	2 (6.5)
Cavitating nodular opacities	1 (3.2)
Cavitary lesion	1 (3.2)
Subpleural lesions with small central necrotic areas	1 (3.2)
Multiple lymphadenopathy	1 (3.2)

**Table 2:** Potential induced-factors of acute fibrinous and organizing pneumonia.

Potential induced-factors	Ref	Steroid effective	Age, years	Gender	Chest CT imaging	Respiratory symptoms
Infection disease(*n* = 7)						
*Aspergillus niger* infection	(2)	Not use/dead	59	Female	Consolidation	Cough, hemosputum, slight fever, anorexia, and malaise
Fungal infection including P. citrinum	(3)	Yes	67	male	Consolidation, ground glass opacities	Cough, fever, dyspnea and hemoptysis
Influenza A and H1N1 infection	(5)	Yes	71	Male	Consolidation and crazy paving.	Fever, cough, dyspnea, and weight loss
Chronic active epstein–barr virus infection	(4)	Yes	64	Male	Patchy consolidation in both lungs and multiple lymphadenopathy in the mediastinum and hilum.	Fever and dyspnea
Pulmonary tuberculosis	(6)	Yes	44	Male	Consolidation and ground glass opacity	Yellow mucous sputum for over 1 month and high fever
Legionella pneumonia (and Sjogren’s syndrome)	(7)	Yes	47	Male	Patchy consolidation	Fever, expectoration, and shortness of breath
Periorbital MRSA cellulitis and rheumatoid arthritis	(8)	NO/dead	78	Male	Ground-glass opacities	Fever with dyspnea
Hematological diseases (*n* = 5)						
Primary mediastinal B-cell lymphoma	(9)	Yes	38	Female	Ground-glass opacity and infiltration (consolidation)	Fever
Chronic myeloid leukemia	(5)	Yes	51	Female	Cavitating nodular opacities	Right-hand pain, erythema, swelling, fever, and cough
Myelodysplastic syndrome	(10)	NO/dead	38	Male	Nodules and consolidation	Fever
Myelodysplastic syndrome	(11)	Yes	72	Female	Parenchymal consolidation	Fever and productive cough
Myelodysplastic syndrome	(12)	Yes	49	Female	Consolidation and ground-glass opacities	Fever, cough, and dyspnea
Myelodysplastic syndrome	(9)	Yes/ Dead of MDS without AFOP relapse.	63	Male	Infiltration (consolidation) and micronodules	Fever
Non Hodgkin’s lymphoma	(13)	NO/ improved with Bendamustine-rituximab	65	Male	Patchy consolidation	Dry cough, dyspnea, anorexia and night sweats (swelling)
Connective tissue disease(*n* = 3)						
Systemic lupus erythematosus	(14)	NO/improved with Cyclophosphamide	25	Female	Diffuse finely granular shadows are evident in both lungs	Fever, cough, and erythema of the face and extremities.
Systemic lupus erythematosus	(15)	Yes	54	Female	Multiple consolidations, predominantly in the lower lobes	Fever
MD5 + dermatomyositis	(5)	NO/dead	52	Male	Diffuse ground-glass changes	Polyarthralgia, mouth ulcers, weight loss, vasculitis rash, and proximal muscle weakness
Anti-cancer drug treatment (*n* = 3)						
Her2-neu–negative breast carcinoma with chemotherapy with epirubicin and cyclophosphamide	(16)	Not use/dead	64	Female	Ground-glass opacities and thickening of the interlobular septal walls.	Cough and dyspnea
Prostate cancer with bone metastasis	(17)	Yes	65	Male	Consolidations and ground-glass density patchy opacities	Fever and chills, exertional dyspnea, and dry cough
Pembrolizumab for progressive urothelial carcinoma	(18)	NO/improved with Tacrolimus	72	Male	Consolidation,	dyspnea
Others (*n* = 4)						
Crohn’s disease	(19)	Yes	22	Female	Solid nodules	Asymptomatic
COVID-19 vaccination	(20)	Yes	42	Female	Patchy consolidation and ground-glass opacity	Fever, muscle pain, and dyspnea
Flecainide	(21)	NO/dead	61	Female	Ground-glass opacities and septal thickening	Dyspnea and dry cough
Lung transplantation	(22)	Yes	33	Female	Consolidations	Fever, cough, and dyspnea
None (*n* = 7)						
None	(23)	NO/azithromycin	52	Female	Cavitary lesion and consolidation	Fever, night sweats(swelling), cough, chest pain
None	(15)	Yes	53	Female	Multiple consolidations, predominantly in the lower lobes	Fever, dyspnea, and cough,
None	(24)	Yes	53	Female	Patchy consolidation	Fever, cough and dyspnea
None	(25)	Yes	55	Male	Sub pleural lesions in bilateral lower lobes, some of which had small central necrotic areas.	Right subcostal pain
None	(26)	Yes	58	Female	Multiple patchy shadows in both lungs (consolidation)	Chest tightness, dyspnea, cough and sputum
None	(27)	Yes	70	Male	Consolidation and “ground-glass”	Fever cough
None	(27)	Yes	71	Female	Ground glass opacification (GGO) and haze,	Dyspnea, fever persisted and sputum thickened

Corticosteroids served as the cornerstone of therapy in over 96% of the cases. The overall efficacy rate of glucocorticoid therapy exceeded 73%, yielding complete or partial radiological and clinical resolution. For cases refractory to corticosteroids, second-line immunosuppressive agents—including cyclophosphamide, bendamustine-rituximab, or tacrolimus—were successfully employed.

## Discussion

AFOP represents a histopathological pattern of severe lung injury that lies within the spectrum of organizing pneumonia (OP) and diffuse alveolar damage (DAD). However, its clinical course, prognosis, and therapeutic responses differ significantly from these entities. While the clinical and radiological manifestations of AFOP are often indistinguishable from conventional infectious pneumonia, its histopathological features are distinct. The pathognomonic finding is intra-alveolar “fibrin balls” with associated organizing tissue, lacking both the classic hyaline membranes of DAD and the predominant intra-broncholar Masson bodies of OP (2).

The pathogenesis of secondary AFOP involves an aberrant, dysregulated inflammatory cascade triggered by various alveolar insults. Our literature review underscores the etiological diversity of AFOP, which includes autoimmune, neoplastic, drug-induced, and infectious etiologies. Notably, fungal infections, particularly *Aspergillus*, are exceptionally rare triggers. In our case, *Aspergillus fumigatus* was isolated directly from the biopsied lung tissue, confirming it as the primary biological driver of the host’s aberrant fibrinoid inflammatory response.

A highly unique and life-threatening complication observed in our patient was the development of spontaneous subcutaneous and mediastinal emphysema. The development of mediastinal emphysema in AFOP likely occurs via the *Macklin effect*: severe intra-alveolar inflammation and fibrin deposition lead to increased alveolar pressure, alveolar rupture, and subsequent dissection of air along the bronchovascular sheaths into the mediastinum and subcutaneous tissues. The high intra-thoracic pressure generated by persistent coughing further exacerbates this process. Prompt recognition and negative-pressure closed thoracic drainage were instrumental in preventing tension pneumomediastinum and cardiovascular compromise in this patient.

Currently, consensus guidelines for the treatment of AFOP are lacking. Systemic glucocorticoids remain the primary therapeutic option, with our review demonstrating a response rate of over 73%. However, the optimal dosage, route of administration, and taper duration remain controversial. In secondary AFOP, addressing the underlying trigger is equally paramount. In this case, combining systemic methylprednisolone with targeted intravenous-to-oral voriconazole therapy successfully arrested the inflammatory process while simultaneously eradicating the underlying fungal infection, leading to complete clinical resolution without disease recurrence.

The predominant symptoms of AFOP, as also reported in the literature (4), include cough, fever, and dyspnea. Less common symptoms such as swelling, erythema, chest pain, weight loss, anorexia, and other extrapulmonary manifestations were observed less frequently. Chest CT scans in most AFOP cases typically reveal consolidation alongside ground-glass opacities. In contrast, a smaller number of patients displayed findings such as nodules, septal thickening, cavitating lesions, subpleural abnormalities with small central necrotic areas, multiple lymphadenopathies.

## Conclusion

This report presents a case of *Aspergillus fumigatus*-induced AFOP, complicated by spontaneous subcutaneous mediastinal emphysema. Based on our literature review, *Aspergillus* as a pathogen of AFOP is exceedingly rare. Among the 31 patients with AFOP included in our review, only one case was attributed to *Aspergillus* infection, corresponding to a frequency of 3.2% (1/31). The case was notable for its relentless progression, lack of association with secondary diseases, and favorable response to glucocorticoid therapy. Our case was diagnosed by biopsy. Sampling limitation may lead to misdiagnosis because small biopsy tissues might not represent the intrinsic lesions. Additionally, we conducted a comprehensive review of AFOP cases reported over the past 5 years. Given the uncertain etiology and the diverse clinical presentations, it is implied that the actual incidence rate may exceed current estimates in the literature.

Therefore, for imaging-diagnosed pneumonia patients presenting with recurrent fever and cough, who respond poorly to intensive anti-infective treatment, the possibility of AFOP should be considered. And the significant clinical insights highlighted in this report should prompt further investigation into AFOP and its underlying mechanisms.

## Data Availability

The original contributions presented in the study are included in the article/supplementary material, further inquiries can be directed to the corresponding author.
